# Nanoscale borosilicate bioactive glass for regenerative therapy of full-thickness skin defects in rabbit animal model

**DOI:** 10.3389/fbioe.2023.1036125

**Published:** 2023-05-18

**Authors:** Noha Elshazly, Manal M. Saad, Rania M. El Backly, Ayat Hamdy, Marco Patruno, Samir Nouh, Suman Saha, Jui Chakraborty, Mona K. Marei

**Affiliations:** ^1^ Tissue Engineering Laboratories, Faculty of Dentistry, Alexandria University, Alexandria, Egypt; ^2^ Department of Comparative Biomedicine and Food Science, University of Padova, Legnaro, Italy; ^3^ Oral Biology, Faculty of Oral and Dental Medicine, Ahram Canadian University, Giza, Egypt; ^4^ Endodontics, Conservative Dentistry Department, Faculty of Dentistry, Alexandria University, Alexandria, Egypt; ^5^ Department of Surgery, Faculty of Veterinary Medicine, Alexandria University, Alexandria, Egypt; ^6^ Bioceramics and Coating Division, Central Glass and Ceramics Research Institutes, Kolkata, India; ^7^ Department of Removable Prosthodontics, Faculty of Dentistry, Alexandria University, Alexandria, Egypt

**Keywords:** bioactive glass, skin regeneration, wound healing, angiogenesis, nanofibers, electrospinning, diabetes mellitus

## Abstract

Bioactive glass (BG) occupies a significant position in the field of hard and soft tissue regeneration. Different processing techniques and formulas have been introduced to expand their regenerative, angiogenic, and antibacterial properties. In the present study, a new formula of bborosilicate bioactive glass nanofibers was prepared and tested for its wound-healing efficacy in a rabbit animal model. The glass formula ((1–2) mol% of B_2_O_3_ (68–69) mol% of SiO2, and (29–30) mol% of CaO) was prepared primarily by the sol-gel technique followed by the electrospinning technique. The material was characterized for its ultrastructure using scanning electron microscopy, chemical composition using FTIR, and its dynamic *in vitro* biodegradability using ICP-AES. Twelve rabbits were subjected to surgical induction of full-thickness skin defects using a 1 cm^2^ custom-made stainlessteel skin punch. The bioactive glass nanofibers were used as a grafting material in 6 experimental rabbits, while the defects in the remaining rabbits were considered as the negative control samples. All defects were assessed clinically for the decrease in wound size and clinical signs of healing and histologically for angiogenesis, collagen density, inflammatory response, cell recruitment, epithelial lining, and appendages at 1,2 and 3 weeks following the intervention. Structural analysis of the glass fibers confirmed their nano-size which ranged from 150 to 700 nm. Moreover, the chemical analysis confirmed the presence of SiO_2_ and B_2_O_3_ groups within the structure of the nanofibers. Additionally, dynamic biodegradation analysis confirmed the rapid degradation of the material starting from the first 24 h and rapid leaching of calcium, silicon, and boron ions confirming its bioactivity. The wound healing study of the nanofibrous scaffold confirmed its ability to accelerate wound healing and the closure rate in healthy rabbits. Histological analysis of the defects confirmed the angiogenic, regenerative and antibacterial ability of the material throughout the study period. The results unveil the powerful therapeutic properties of the formed nanofibers and open a new gate for more experimental and clinical applications.

## 1 Introduction

Bioactive glass-a third-generation biomaterial-has occupied a significant position in many clinical applications of tissue engineering, regenerative medicine, and dentistry fields throughout the last 2 decades up till now (Jones, 2015; Montazerian and Zanotto, 2017; Carvalho et al., 2019). The gold standard 45S5 bioactive glass was mainly invented to target bone regeneration through initiating bone formation in its biological environment (Hench, 2006; Jones, 2015). However (Wilson et al., 1981) were the first to prove its ability to contact and promote soft tissue regeneration as well. It prompts specific responses on the molecular level of cells, leading to the initiation of *in situ* tissue regeneration (Bellucci et al., 2019) (Sergi et al., 2020). The bioactivity of bioactive glass is mediated by the formation of the hydroxycarbonate apatite layer (HCA) on the surface of bioactive glass when it contacts body fluids. This formed layer allows the bioactive glass to form a stable bond with soft and hard tissues without fibrous tissue formation (Jones, 2015) (Hench, 2006).

Throughout the years, additional types of bioactive glass have been developed and involved different kinds of therapeutic ions such as zinc, silver, copper, sodium, and boron ions (Rahaman et al., 2011; Jones, 2015). Boron-containing bioactive glass gained special attention due to its ability to enhance glass bioactivity and tissue reaction (Mazzoni et al., 2021). Indeed, the incorporation of borate ions influences the regenerative potentiality of living tissues (Mazzoni et al., 2021). Furthermore, the bioactivity levels of borate or borosilicate bioactive glass exceed that containing silicate ions alone (Huang et al., 2006). For that reason, it reacts with surrounding body fluids in a faster pattern and converts more rapidly to the hydroxy carbonate apatite layer (HCA)allowing rapid tissue reaction and regeneration (Jung and de, 2009; Bi et al., 2013). In context with that, borate-containing bioactive glass has the upper hand when targeting soft tissue regeneration (Rahaman et al., 2011).

The regenerative efficacy of bioactive glass as a single scaffold or in combination with cells or other scaffolds such as collagen and chitosan has been reported (Baino et al., 2018; Kargozar et al., 2019). Indeed, adding BG to polymers and other biocomposites stimulates wound regeneration and diminishes the infection at the implantation site (Sarker et al., 2015; Nour et al., 2019; Sergi et al., 2020)**.** This potentiality is highly related to the ionic dissolute and the ultra-structure of the bioactive glass (Sergi et al., 2020). Certainly, it is well known that BG leaching ions regulate gene expression, cell apoptosis, progenitor cell migration, proliferation, and differentiation at the wounding area (Labbaf et al., 2011; Gong et al., 2012). Moreover, they influence angiogenesis and the formation of a fibrin clot, collagen, and epithelial tissue regeneration (Lin et al., 2014; Zhao et al., 2015). For instance, the leached silicate and borate ions have a significant role in angiogenesis by allowing endothelial cell proliferation and the formation of endothelial tubules (Zhao et al., 2015). Moreover, they increase the secretion of vascular endothelial growth factor (VEGF) and its gene expression in fibroblast cells (Li and Chang, 2013; Kargozar et al., 2018). In addition to that, borate ions also help in the regulation of extracellular matrix (ECM) and boost the collagenase and cathepsin D activity in fibroblast cells (Nzietchueng et al., 2002; Pizzorno, 2015). In concomitant with that, calcium ions are well known for their role in initiating hemostasis at the early stage of wound healing. Besides, they diminish the inflammatory response and accelerate epidermal cell regeneration, proliferation, and migration (Kargozar et al., 2019). Also, they contribute to the antibacterial action of bioglass through bacteria calcification and raising the alkaline PH at the injury site (Ma et al., 2014; Naseri et al., 2017; Kargozar et al., 2019). Thus, bioactive glass expends its therapeutic effect on all three stages of wound healing allowing it to culminate in wound regeneration.

The ultra-structure Bioactive glass also influences its regenerative ability, as the higher surface area is expected to increase the material bioactivity through increasing solubility rate, resulting in faster ions leaching, and protein adsorption (Hench and Polak, 2002; Hench, 2009; Hajiali et al., 2010; Sergi et al., 2020). For that reason, porous nanoscale bioactive glass provides a suitable environment for soft tissue regeneration (Ma et al., 2014; Zhao et al., 2015). This mimics the natural extracellular matrix (ECM) and results in the enhancement of cell proliferation and migration (Xu et al., 2015). Moreover, its huge surface area and a multiscale large percentage of porosity and interconnective pores grant cell growth and spreading (Webster et al., 2000; Xu et al., 2015). Besides, it indorses cell communication with the surrounding environment which promotes the flow of nutrients and growth factors toward cells and the waste products outside the cells (Peter, 2011; Ma et al., 2014). Among different techniques used to produce nanofibers, electrospinning is a simple, low cost and controllable technique able to produce contentious and diameter controllable bioactive glass scaffold mimicking ECM (Kim et al., 2006; Ma et al., 2014; Deliormanlı, 2015).

Our group has previously confirmed the regenerative ability of borate-based bioactive glass nanofibers (BGNF) composed of (1–2) mol% of B2O3 (68–69) mol% of SiO2, and (29–30) mol% of CaO in promoting oral mucosal regeneration in diabetic rabbits (Elshazly et al., 2020). The used nanofibers-initiated reepithelization of wound defects as early as 1 week after grafting. Moreover, they stimulated angiogenesis by increasing VEGF production levels and stasis of bacterial growth at the wounding area.

As the mucosal tissue has a higher potentiality to wound healing when compared to the skin tissue, the present study aimed at preparing, characterizing, and evaluating the same formula of borate bioactive glass nanofibers on full-thickness skin wounds created in normal rabbits. We investigated the fibers’ regenerative, angiogenic, and antibacterial properties using different means of clinical and histological analysis.

## 2 Materials and methods

### 2.1 Bioactive glass preparation

**FIGURE 1:**
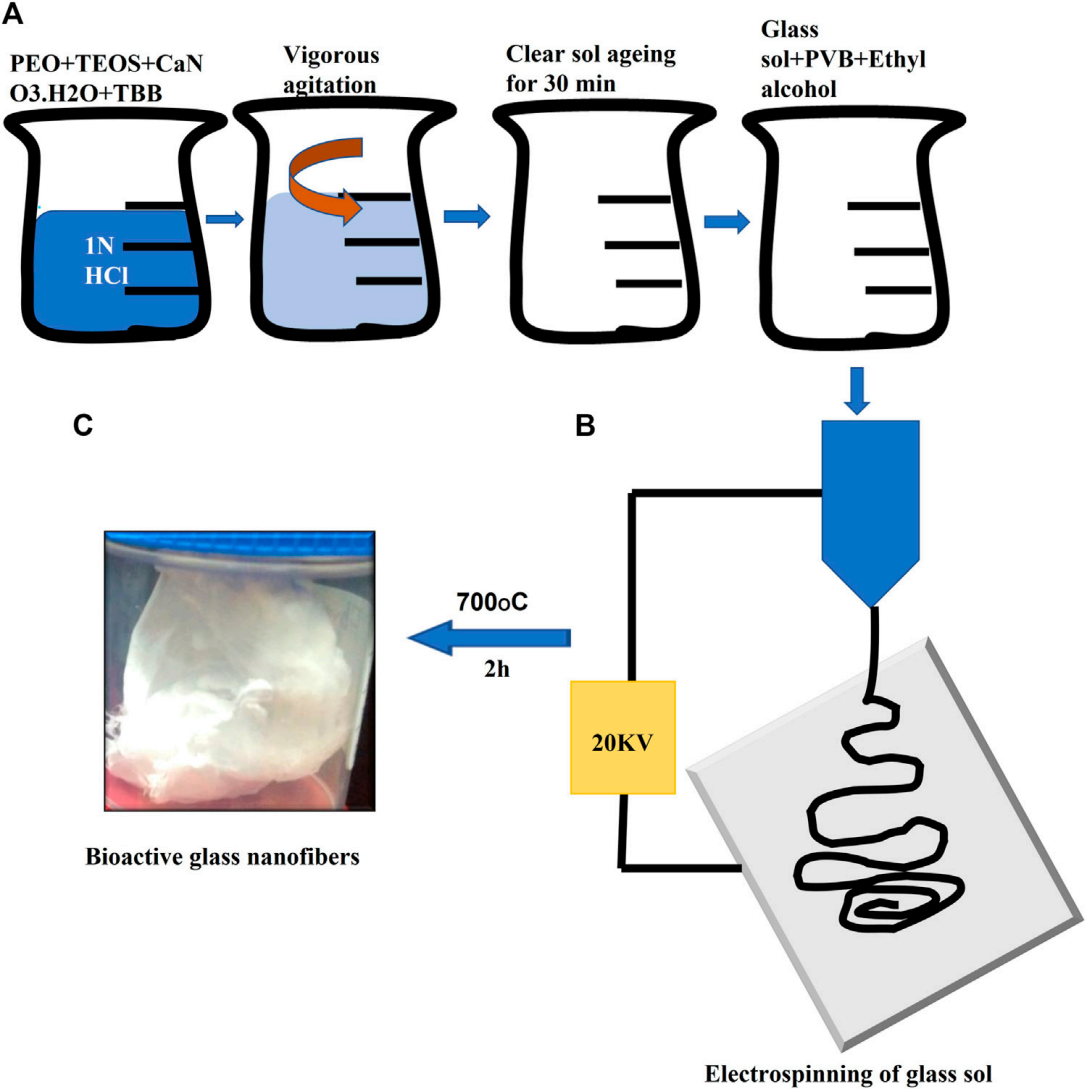
Illustration of borate bioactive glass nanofibers preparation. Where **(A)** are serial steps for preparation of glass sol and formation of glass/polymer mixture to control sol viscosity. **(B)** Diagram of the electrospinning process. **(C)** Image of the final formed borate bioactive glass nanofibers after heat treatment.

Borate-based bioactive glass nanofibers of composition (**(1–2) mol% of B**_**2**_ **O**_**3**_ **(68–69)mol% of SiO**_**2**_ **(29–30)mol% of CaO) **were prepared using the low-temperature sol-gel technique as previously described by our group in the following sequence: addition of polyvinyl butyral (PVB), Tetra Ethyl ortho silicate (TEOS), Tributyl borate (TBB) and Calcium nitrate tetrahydrate (CaNO_3_.4H_2_O) (Sigma Aldrich, Bangalore, India), was done in a 1N HCl solution for the preparation of glass sol. The glass sol was mixed with a 1.8% polyvinyl butyral polymer solution (70% ethanol solution) in a ratio of 3:2 in order to improve glass sol rheological properties. Following that, Bioactive glass nanofibers were prepared by electrospinning glass sol/polymer mixture using a nanofiber electrospinning unit (Nano NC, Seoul, Republic of Korea). During this procedure, a 15 cm glass syringe with an 18 gauge metallic needle was charged with the glass/polymer mixture. The electrospinning unit was operated under 18 kV DC voltage, with sol injected speed of 0.4 mL/h. At about 5 cm from the capillary, the nanofibers were gathered on Teflon-coated aluminum foil that was put on a plate connected to the device. All the previous procedures were done at The Central Glass and Ceramic Research Institute (CGCRI), Kolkata, India and fully described by (Elshazly et al., 2020; Saha et al., 2020) (Figure 1).

### 2.2 Glass nanofibers characterization

Glass nanofibers were characterized for their diameter, porosity and crosslinking using transmitted scanning electron microscopy **(**JEOL 7900F, Japan) (Elshazly et al., 2020). Analysis of chemical bonds of the formed nanofibers was performed using Fourier Transform Infrared Spectroscopy (FTIR) (Bruker Equinox 55) (Saha et al., 2020). The spectra in absorbance mode were recorded using the KBr pellets. The resolution used was 4 cm-^1^, and the functional groups obtained were within the wave number range of 4,000–400 cm-^1^ (Deliormanlı, 2015). Finally, Dynamic *in vitro* biodegradation of the glass fibres was done by soaking BGnf at a concentration of 0.0004 g/ml at 37°C in simulated body fluid (SBF) under contentious stirring to simulate the dynamic nature of the body fluids (Figures 3C, E) (Saha et al., 2020). Analysis of the BG extract was done at 24, 48, and 150 h time intervals. The BGs extract was analyzed using inductively coupled plasma atomic emission spectroscopy (ICP-AES) (Spectro Analytical, Kleve, Germany) to determine the amount of B, Si, and Ca ions released in the SBF.

### 2.3 Sterilization of the glass fibres

The bioactive glass nanofibers were primarily sterilized by gamma rays followed by vacuum sealing. At the time of experiments, BGnf were re-sterilized under UV light (Wavelength of 200–280 nm) for 2 h (Yang et al., 2015; Dai et al., 2016).

### 2.4 *In vivo* wound healing study

#### 2.4.1 Animals

Twelve Male New Zealand white rabbits with an average age of 2, 3 months and weighing about 2.5–4 Kg were utilized in the *in-vivo* studies. The animals were housed in separate standard cages and the standard amount of pelleted food (133 g daily), as well as freshwater, was supplied. Rabbits were subjected to equal cycles of daylight and dark. The standard temperature ranged from 10°C in winter to 30°C in summer. All the experiments followed the NIH guidelines for animal care and welfare (National Research Council, 2011). Experiments were approved by the Institutional Animal Care and Use Committee of Alexandria University, Egypt, a member of the International Council for Laboratory Animal Science (ICLAS30.1.2019). All studies were performed under standard sterile conditions.

#### 2.4.2 Effect of BGnf on full-thickness skin wound healing in healthy rabbits

**FIGURE 2:**
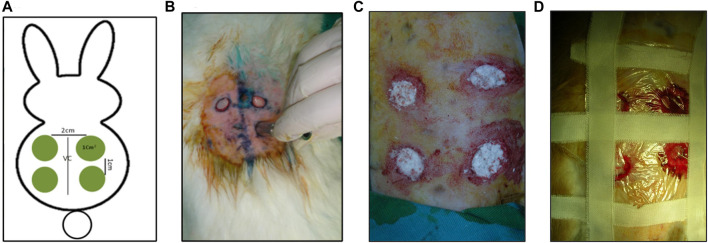
Clinical pictures of the Creation of full-thickness skin defects. **(A)** Diagrammatic illustration of the full thickness skin defects and their position on rabbit back. **(B)** Creation of full-thickness skin defects using a custom-made stainless-steel punch. **(C)** Four full-thickness skin defects were grafted with 0.07–0.09 g of bioactive glass nanofibers. **(D)** Skin wounds covered with Tegaderm.

Based on the previous study conducted by our group on the oral mucosa, the experimental and control wounds were separated into two different groups of rabbits. The separation was aimed at avoiding the indirect effect of the BGnf graft by leaking to the subcutaneous tissues of the other groups of defects. A total of 12 rabbits were randomly and equally divided into a sham control (*n* = 6) and an experimental group (*n* = 6) with a total number of 36 defects. For each rabbit, the back was shaved, and the rabbit was anesthetized by intramuscular administration of xylazine HCL in a dose of 5 mg/kg (Sigma-tech pharma industries, sixth of October City, Egypt) followed by ketamine in a dose of 50 mg/kg (Rotexmedica, Trittua, Germany). After that, the back was then disinfected using betadine. Following that, full-thickness skin defects of 1 cm^2^ were created on either side of the rabbit’s back using a custom-made stainless-steel circular skin punch (Dong et al., 2017) (Figures 2A,B). For the experimental group, the full thickness defects (*n* = 18) were grafted with a single application of 0.07–0.09 g of borate-based bioactive glass nanofibers (Figure 2C). On the other hand, defects of the sham control group (*n* = 18) were left untreated for the whole study period. The wound areas were then covered with Tegaderm (TM, 3M- Healthcare, Germany) (Figure 2D). Post-operative analgesic and antibiotic cefotaxime (Cefotax 1g, Egyptian. Int. Pharmaceutical (E.I.P.IC.O).10th of Ramadan city. Egypt) in a dose of 150 mg\kg\day and ketorolac (Ketorolac, AmryaPharm.IND., Alexandria—Egypt) in a dose of 60 mg\day were given to rabbits for 3 days after surgery (Dong et al., 2017).

#### 2.4.3 Clinical scoring and wound closure rate

All groups of wounds were photographed on days 0,5,7,14, and 20 at a standardized distance of 10 cm using a digital camera (Sony DSC-T7) and a gross clinical scoring and assessment were done by two blinded researchers following the scoring criteria presented in (Table 1) (Fletcher, 2010; Lin et al., 2012). Furthermore, the decrease in wound area was measured at the same time intervals using ImageJ software (Li et al., 2022). The progress in the percentage of wound area reduction over different time intervals was calculated according to the following equation (Thomas and Wysocki, 1990; Li et al., 2022):

$$Wound\;healing\;rate=\left(A0-A1\right)\times 100/\;A0$$

**TABLE 1:** Demographics of animal model.

Total N	Study groups		Nu of defects	
	Condition	N	Condition	
12 New Zealand white rabbits	Control	6	Control	18
	Healthy experimental	6	Healthy experimental	18

Where A0: The initial wound surface area. A1: The wound surface each day.

#### 2.4.4 Tissue preparation and histological staining

Rabbits were euthanized at 1-, 2- and 3-week intervals using an overdose of xylazine, and ketamine followed by cervical dislocation (Gail, 2013). The wound area and 1 cm^2^of surrounding intact skin were harvested (Lin et al., 2012; Zhao et al., 2015). For histological evaluation, the samples were subjected to fixation in formalin 10% then samples were dehydrated in graded series of different concentrations of ethanol (70%, 80%, 90% and 100%). Dehydrated samples were cleared using xylene, embedded in paraffin wax, and were then cut into 7 µm thin sections. Embedded sections were stained with haematoxylin and eosin (H&E) (Biotec, Chelopech,Bulgaria) and then evaluated and photographed by light microscopy (Olympus, Tokyo, Japan) (Elshazly et al., 2020). Table 2 For Gomori trichrome staining; sections were primarily stained with Haematoxylin, rinsed with water and soaked in the trichrome stain for 25 min. Following that sections were rinsed twice in glacial acetic acid (0.2%) for 1 min each. Finally, sections were dehydrated with alcohol 100%, then xylene and mounted to be evaluated and photographed using light microscopy (Olympus, Tokyo, Japan) (Zaki, 2015).

**TABLE 2:** Scoring of macroscopic assessment of wound healing.

Variables		Scores
Exudates	1	No Exudate
	2	Mild
	3	Moderate
	4	Abundant
Moisture level	−2	Dry++
	−1	Dry+
	0	Normal
	1	Moist wet
	2	
Tissues in the wound bed	1	Absent
	2	Mild
	3	Moderated
	4	Abundant
	5	Healed skin

#### 2.4.5 Histomorphometric analysis

Analysis of epithelial thickness was performed using ImageJ software (NIH, United States), where a skilled histologist blindly evaluated the analyses of tissue slices by choosing three random, non-overlapping areas at a magnification of ×10 per tissue section. The epithelial gap as well as percentage of reduction in wound size were measured using four panoramic images of different histological sections following the protocol described by Hou et al. (2022).

For collagen density measuring, random areas (four-five) at a magnification of ×10 of trichrome stained sections of 2 and 3 weeks’ time intervals were blindly assessed using the color threshold method of the ImageJ software following the protocol described by Hong et al. (2020) (Figures 8A, B).

Blood vessel density at different time intervals was measured by a new software called IKOSA software (KML Vision GmbH, 8,020 Graz, Austria). Briefly, a number of (20–25) images per each time interval were uploaded to the software, and the blood vessels were annotated by a blind histologist. Following that the software was trained and using the machine learning concept the software was able to determine all the blood vessels in the randomly selected images and calculate the percentage of blood vessels area/total area (Figure 9A).

#### 2.4.6 Statistical analysis

Statistical analysis was carried out by SPSS 16.0 software Both the paired and unpaired t-tests were used to analyse statistical differences. When *p* ≤ 0.05, significance was taken into account. For each and every variable across all groups, means, and standard deviations were calculated. Table 3.

**TABLE 3:** Mean percentage of wound closure rate.

Condition (Mean ± STd)	Time interval					
	0		5	7	14	20
Control	0		17.78±	49.4975±	91.55943±	98.39±
			5.874,932	7.56511	3.278	1.9078
Healthy experimental (BG)	0		*30.672±	*65.2675±	*97.0575±	100± (ns)
			4.075374	6.8377	2.4762	0.43728
*p* values			0.0038	0.0086	0.0173	0.1032

*Statistically Significant *p* ≤ 0.05.

## 3 Results

### 3.1 Structural evaluation of bioactive glass nanofibers

**FIGURE 3:**
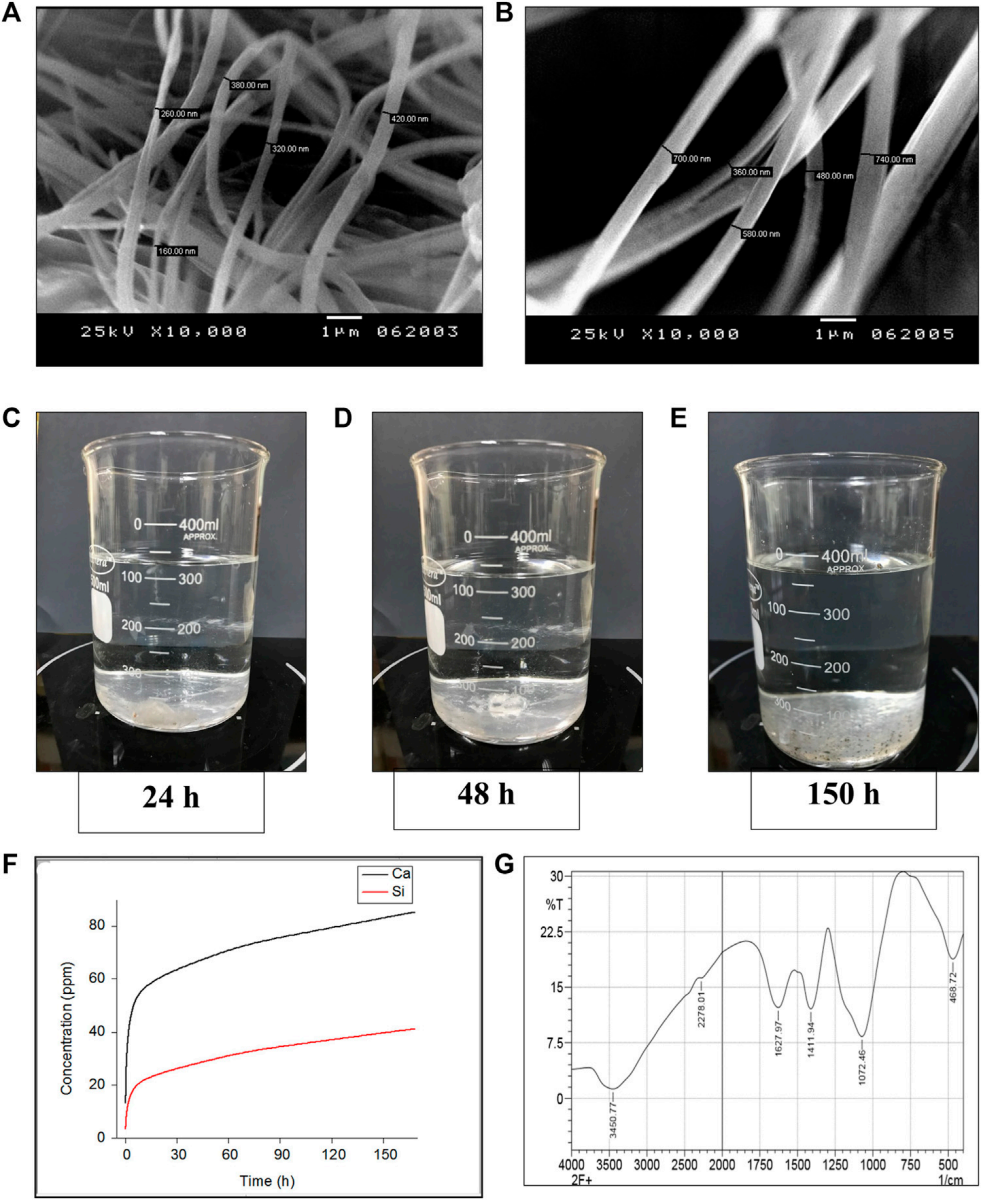
*Invitro* characterization of glass nanofibers **(A, B)** Scanning electron microscopy photographs showing Crosslinked BG nanofibers of a diameter ranging from (150 ‐ 740 nm) **(C–F)** *Invitro* dynamic ionic dissolution of BG nanofibers **(C–E)** Showing gradual degradation of the BG nanofibers soaked in simulated body fluid under stirring at different time intervals (24, 48, and 150 h successively). **(F)** The concentration of the Ca and Si ions dissolute from BG nanofibers soaked in simulated body fluid over 1 week time period shows a gradual increase of both ions across time. **(G)** FTIR analysis of the chemical composition of nanofibers.

Structural characterization of BG nanofibers revealed the presence of crosslinked fibers with multiscale porosity resembling that of the fibrin clot. The fibers’ diameters ranged from 150 nm to 740 nm which confirms the nanoscale range of the formed fibers (Figures 3A, B).

#### 3.1.1 Chemical evaluation

Fourier analysis of the formed BG nanofibers revealed different bands at 3,450, 2,278, 1,627, 1,411, 1,072, and 468.72 (Figure 3G). The bands corresponding to (OH) stretching groups are shown at peaks 3,450 and 1,627 (Jabeen and Rafique, 2014; Abdellaoui et al., 2018). While the band at 2,278 indicated the presence of (Ca (CO_3_)_2_) bond in the formed nanofibers (Abdellaoui et al., 2018). Moreover, the peak at 1,411 is assigned to the (BO_3/2_) bond according to (Abdellaoui et al., 2018) and (Shao et al., 2015). On the other hand, the band at 1,072 can be assigned for asymmetric (Si-O-Si) bond along with borate in the BO_4_group (Shao et al., 2015; Abdellaoui et al., 2018). Furthermore, the chemical analysis revealed a (Si-O-Si) bond at band 468.72 (Jabeen and Rafique, 2014). Chemical analysis confirmed the chemical compositions of the BG nanofibers.

#### 3.1.2 Dynamic biodegradation

Dynamic *in vitro* biodegradation of the glass nanofibers showed a gradual increase of the Si and Ca ions over time reaching their maximum amount after 7 days. The rate of Ca ions leaching in the SBF was double that of the Si ions over the same period as shown in (Figure 3).

### 3.2 *In vivo* wound healing study

#### 3.2.1 Clinical scoring and wound closure rate

##### 3.2.1.1 Clinical assessment of scoring criteria

**FIGURE 4:**
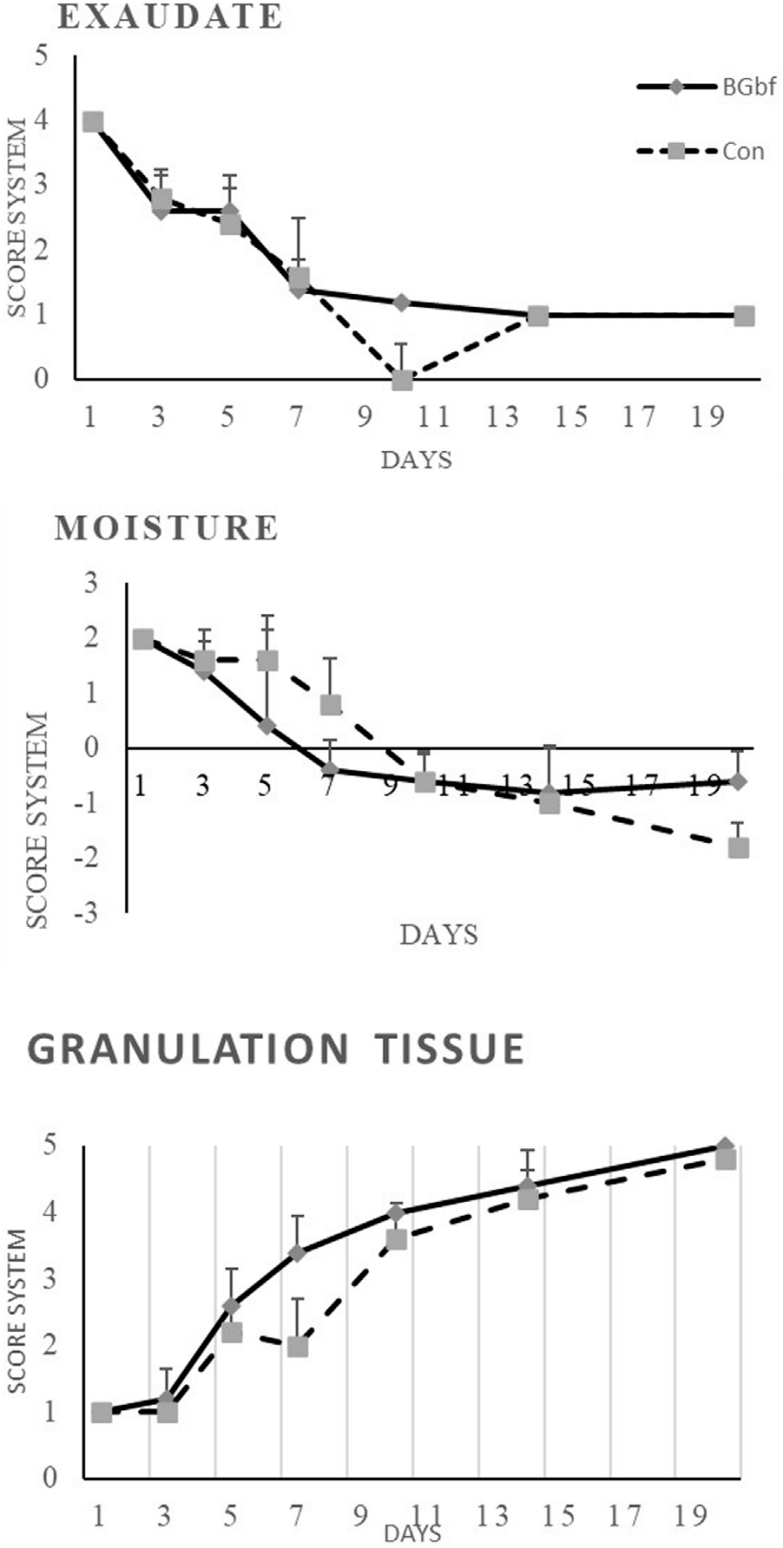
Clinical assessment of full-thickness skin wounds at 1, 2, 3 weeks time intervals using the scoring system mentioned in Table 1 (**(A)** Scoring of wound exudates level of the treated and untreated group of wounds. **(B)** Clinical scoring of the wound moisture level. **(C)** Macroscopic grading of the level of granulation tissue formation.

**FIGURE 5:**
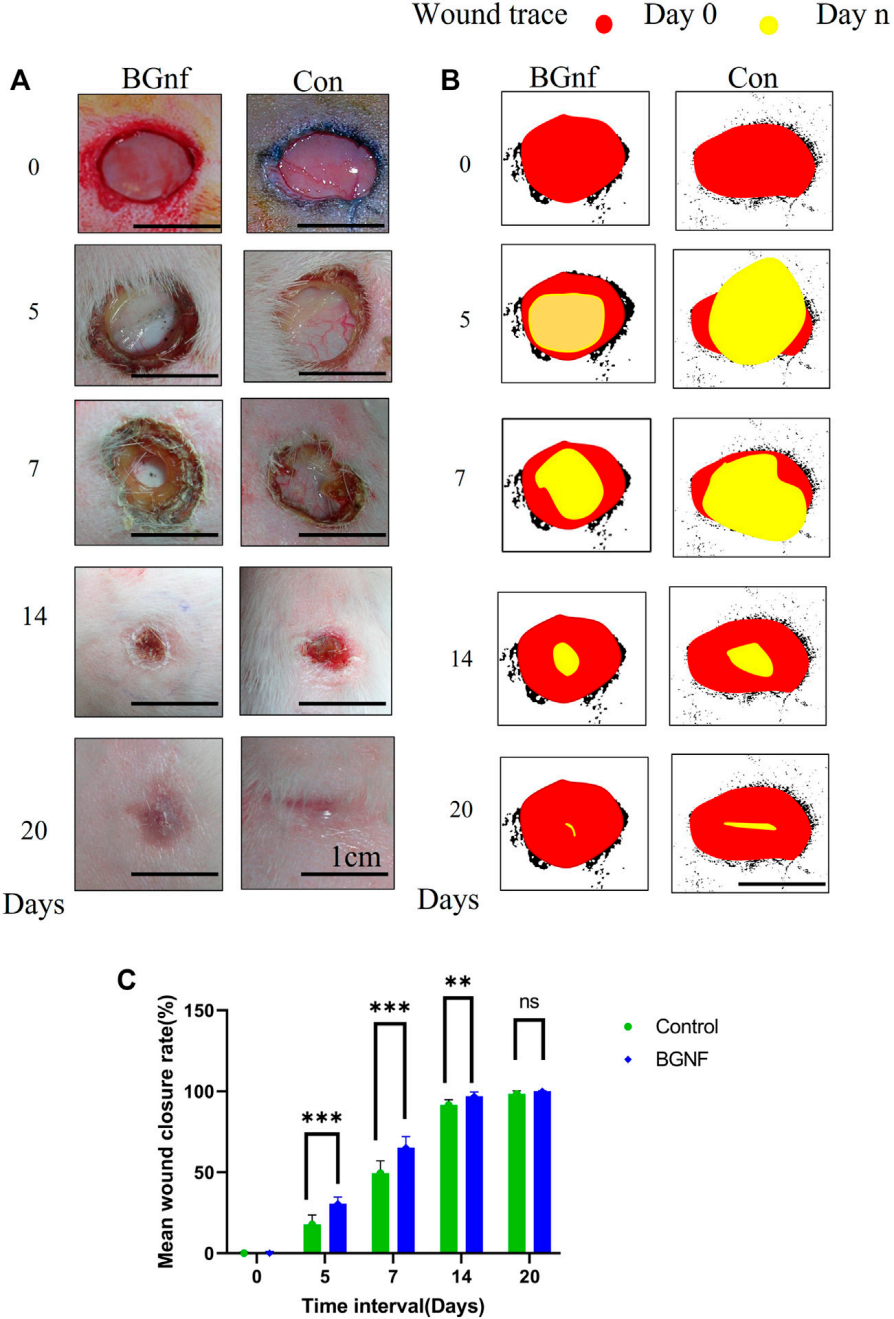
Effect of Borate bioactive glass nanofibers on full-thickness skin wound healing in rabbit model. **(A)** Clinical pictures of the wound areas for different groups (BGnf, Control) on 0, 5, 7, 14, 20 days postoperative. **(B)** ImageJ illustrative diagram of the remaining wound area for the different group *invivo* where the red circle represents the wound area on day 0 and the yellow circle present the wound area in day n. **(C)** Statistical analysis of the percentage of reduction in wound size in different groups at 0, 5, 7, 14, and 20 days postoperative. Values are demonstrated as mean ± standard deviation (*n* = 6) (*p ≤ 0.05).

Clinical scoring within the first week of the skin wounds grafted with borate-based bioactive glass nanofibers showed complete material disappearance from the wound bed and pigmentation at the wound edge after 1 day postoperative. In addition to that, a decrease in wound moisture level, as well as peri-wound erythema, was also observed over time. Tissue creeping from wound edges started from day five postoperative reaching granulation tissue coverage of wound bed and beginning of hair growth around the wound with no signs of infection at the end of the first week were observed (Figures 4, 5A). Within the second-week time intervals, complete re-epithelialization was observed in the wound grafted with borate-based bioactive glass nanofibers when compared to the control wound. At the third week time interval, healed skin of both wounds was observed.

##### 3.2.1.2 Measuring rate of wound contraction

The mean percentage of wound contraction of borate-based bioactive glass nanofibers grafted wounds, as well as control wounds, were fit into several curves representing the wound contraction rate as a function of healing days as shown in the graph in Figure 5C. Borate-based bioactive glass nanofibers grafted wounds showed a statistically significant (*p* = 0.0086) contraction rate reaching 65.3% of wound size at a 1-week time interval in comparison to 49.5% contraction of the control wound (Figures 5A–C). The wound contraction rate of the BGnf grafted wounds reached 97.05% at 2 weeks time intervals (*p* = 0.0173) and 100% at 3weeks time intervals (*p* = 0.103) while that of the control wound increased to 91.5% at 2 weeks’ time intervals and 98.3% at 3 weeks’ time intervals. (Figure 5C).

##### 3.2.1.3 Histological examination using H&E stain

**FIGURE 6:**
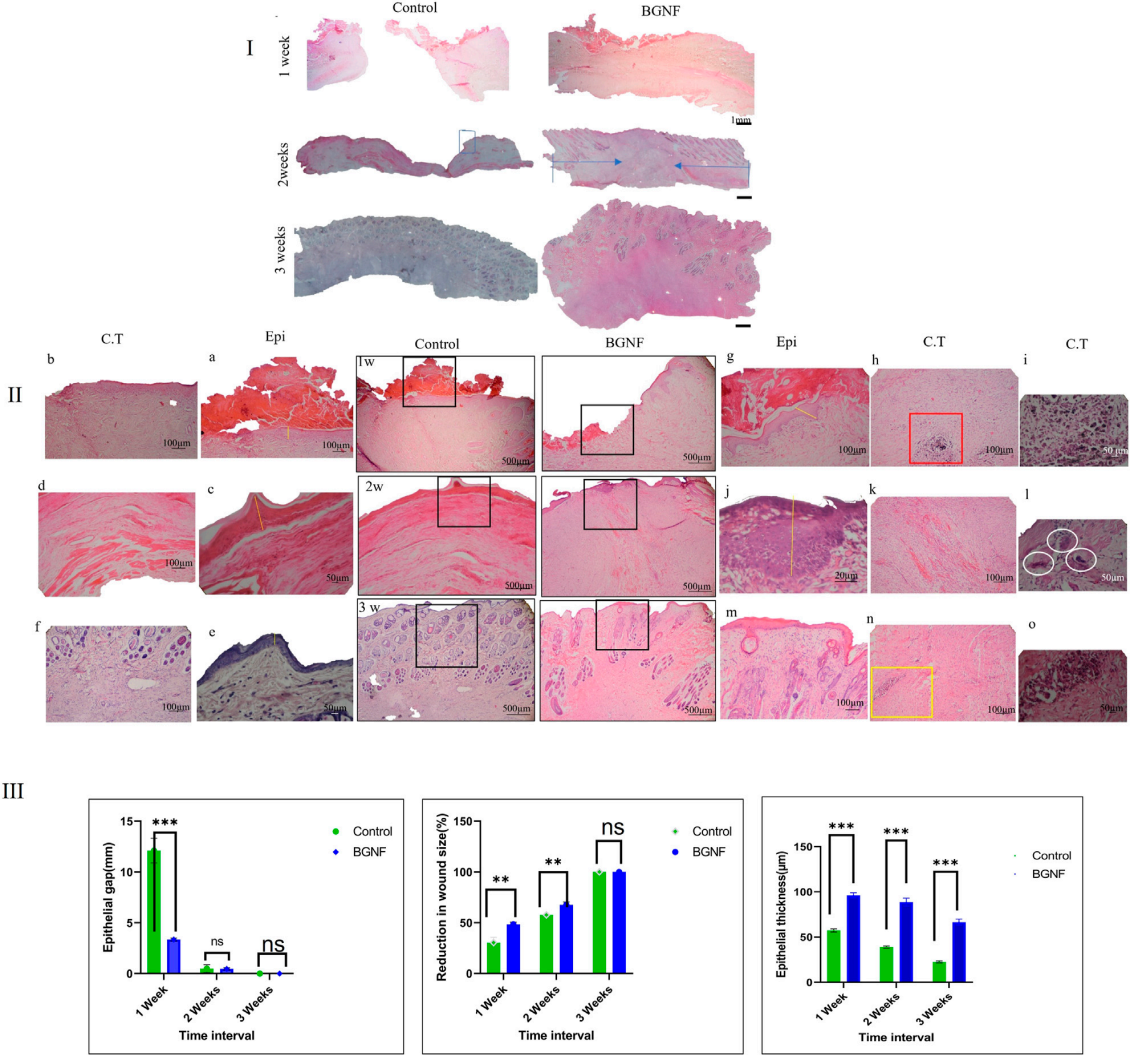
Light Microscope images of H&E stained sections of untreated (control)wounds and BGNF grafted wounds at different time intervals **(I)** Panoramic view of the wounds at different time interval (scale bar = 1 mm) the full width representing the defect while the blue arrow showing the neoepithelization **(II)** Histological images of the untreated and BGNF grafted wounds at higher magnification (scale bar ranges from 500 to 20 µm) giving detailed description of full thickness skin wounds at 1, 2, 3 weeks time intervals **(a, c, e)** images of the epithelial lining of the untreated wound at different time intervals showing the transformation from crust tissue till complete reepithelization **(b, d, f)** light microscope images of the connective tissue lining of the untreated wounds showing the spreading of collagen fibers, blood vessels and fibroblast cells **(g, j, m)** images of the epithelial lining of the BGNF grafted wounds at different time intervals showing the transformation from crust tissue till complete reepithelization while **(h, k, n)** representing a higher magnification of the connective tissue. **(i)** Showing the deep connective tissue layer of the 1-week BGNF treated wound having traces of the glass biomaterial engulfed by multinucleated giant cells and surrounded by newly formed blood vessels (scale bar 20 µm) **(l)** showing multinucleated giant cells (white cycles) in the deep connective tissue layer at 2 weeks time intervals of BGNF treated wounds (scale bar 20 µm)**(o)** images of traces of BGNF in the deep connective tissue layer of BGNF treated wound at 3 weeks time interval confirming the presence of the material throughout the study period **(III)** Measuring of the epithelial gap, microscopic reduction in wound size, and epithelial thickness of the control and BGNF treated groups at different time intervals (the data were presented as mean and standard deviation, (*p* ≤ 0.005).

Histological examination of the control and BGnf grafted wounds in the healthy rabbits expressed differences in the tissue response at 7, 14 and 21 days’ time intervals. The histological results for the different groups of wounds at 7 days are demonstrated in Figure 6. Variabilities in the degree of the inflammatory response, angiogenesis, formation of granulation tissues, cell recruitment, epithelial closure and thickness and arrangement of collagen fibres of both groups were very noticeable. Despite the granulation tissue formed within the wounds treated with BGnf being thinner than that formed within the sham group, it was significantly more cellularized and vascularized (Figure 6II a, g). Moreover, although the collagen fibers were dense disorganized fibers in both groups, they also represented significantly higher cellular infiltration and vascularization when compared to the control wounds (Figure 6II h, b). There was a pronounced inflammatory response in the superficial granulation tissue and deep connective tissue layers in the BGnf grafted wounds. In the deep connective tissue layer, there were inflammatory cells as well as multinucleated giant cells engulfing the remaining BGnf (Figure 6II i). On the other hand, the control wounds showed a regular inflammatory response in both the superficial granulation tissue layer and the connective tissue layer.

The histological results at 14 days postoperatively are shown in Figures 6I, II), BGnf-treated group showed closed thin keratinized epithelial lining with an active basal cell layer (stratum basalis showed deeply stained prominent nuclei (Figure 6II j). The same findings were observed in the control group of wounds although the basal cell layer missed the prominent appearance of the nuclei (Figure 6II c). On the level of the connective tissue layer, BGnf grafted wounds manifested with well-organized horizontally oriented dense collagen fibers with a high cellular distribution. The collagen fibers ran parallel to the overlying epithelium except at the center of the defect where the fibers took a vertical arrangement and then followed the ordinary horizontal direction (Figure 6II k). On the other hand, the control wounds were presented with horizontally oriented collagen fibers that ran parallel to the overlying epithelial lining with a regular cellular distribution (Figure 6II d). Bioactive glass grafted wounds showed excessive dispensation of blood vessels giving an indication of high angiogenic activity (Figure 6II k). In the center of the defect, some newly formed blood vessels took a vertical direction and were surrounded by high cellular activity. It was observed that the pool of the fibroblast-like cells followed the direction of these blood vessels. The control wounds at the same time interval demonstrated a noticeable decrease in angiogenic activity and cell recruitment. The absence of skin appendages was observed in both groups of wounds. Besides, the inflammatory response was mild in both groups. In the deep subcutaneous layer of BGnf treated group traces of BGnf engulfed by multinucleated giant cells were observed (Figure 6II l).

Histological examination of the wounds at 3 weeks is shown in Figures 6I, II). Both groups presented with a thin keratinized epithelial lining that cover the wound area. The connective tissue layer at the BGnf grafted wounds showed densely organized collagen fibers horizontally arranged parallel to the surface epithelium with regular vascular distribution and skin appendages that did not reach the full thickness of the dermal layer. While the control wound presented with loosely organized collagen fibers that ran parallel to the epithelial lining. The connective tissue layer in the control group also manifested with a higher vascular and cellular distribution with skin appendages that did not reach the full thickness of the dermal layer. This gave the indication that the experimental group of wounds were still in their active regeneration phase meaning a dimmish of the future scar tissue formation. Traces of bioactive glass nanofibers were still observed (Figure 6II o).

##### 3.2.1.4 Histological examination using gomori trichrome stain

**FIGURE 7:**
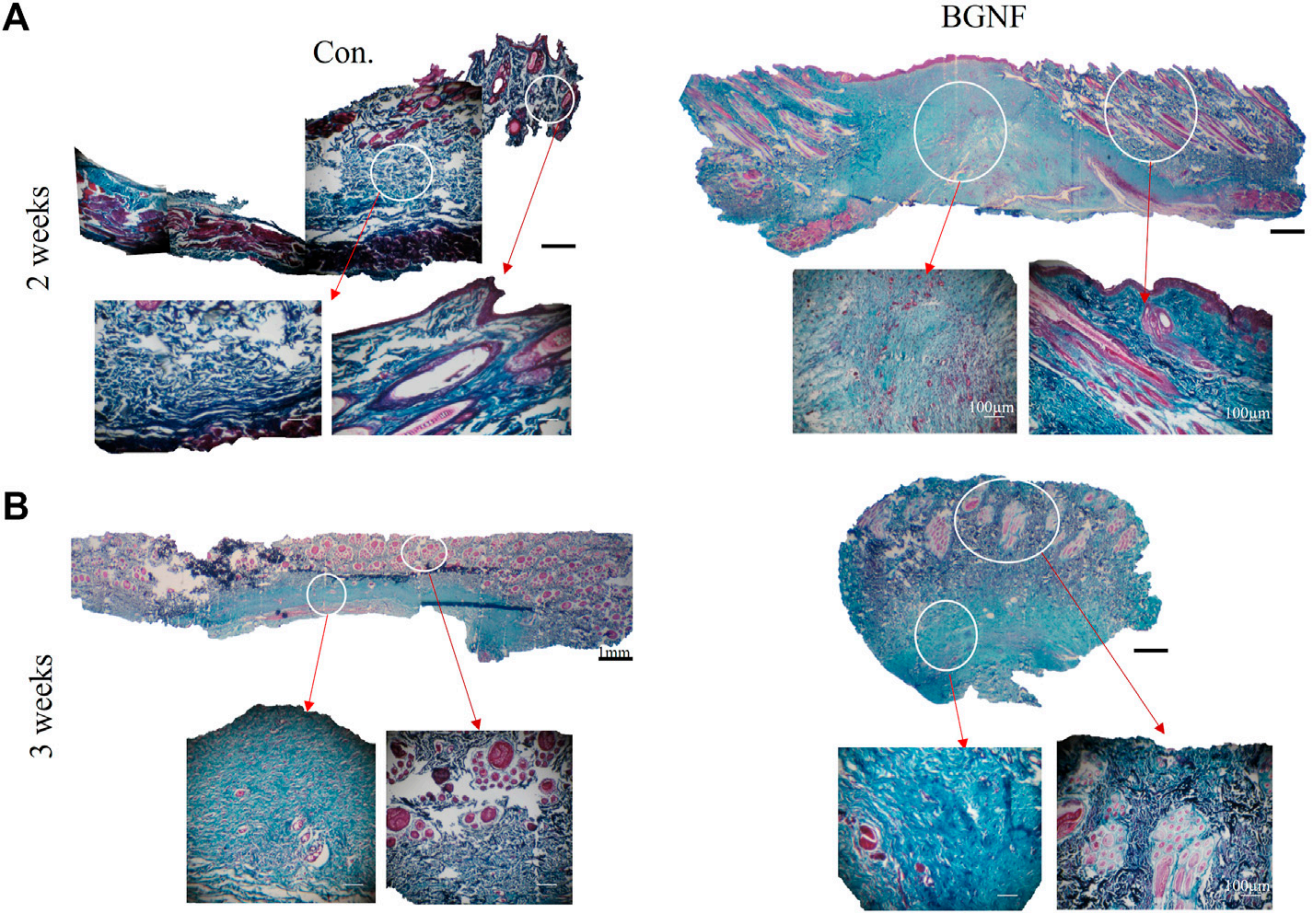
Light Microscope images of Gomori stained sections of untreated and BGNf grafted wounds. **(A)** low (×1) and high magnification (×10)trichrome stained images of untreated and BGNF grafted wounds showing collagen fibers and blood vessels arrangement and density. **(B)** Microscopic images of trichrome stained sections at 3 weeks time interval.

Ttichrome stained sections of 2 and 3 weeks time intervals (Figures 7A, B) revealed well organized densily arranged collagen fibers of the BGnf treated wounds when compared with loosely arranged collagen fibers of control wounds.

##### 3.2.1.5 Histomorphometric analysis of histological sections

Histomorphometric analysis BGnf treated group panoramic images showed a significant decrease in the epithelial gap that reached 3.35 mm when compared with 12.21 mm of the control group at 1 week time interval (*p* = 0.0002). During 2 weeks time interval this gap decreased to reach 0.49 mm in the BGnf treated wounds when compared to 0.49 mm of the control wound which was not statistically significant. On 3 weeks time interval both wounds showed 0 mm epithelial gap (Figure 6III).

When it comes to epithelial thickness, the BGNF treated wound showed the mean epithelial thickness of 96.22 μm when compared to 57.40 µm of the control wound at 1 week time intervals (*p* = 0.0001). At 2 weeks, time interval the mean epithelial thickness of the BGnf treated group decreased to 88.73 µm while that of the control wounds reached to 38.88 µm (*p* = 0.0001). On 3 weeks’ time interval, epithelial thickness of BGnf treated wound was 66.54 µm while that of the control wound was 22.6 µm (*p* = 0.001) (Figure 6III).

**FIGURE 8:**
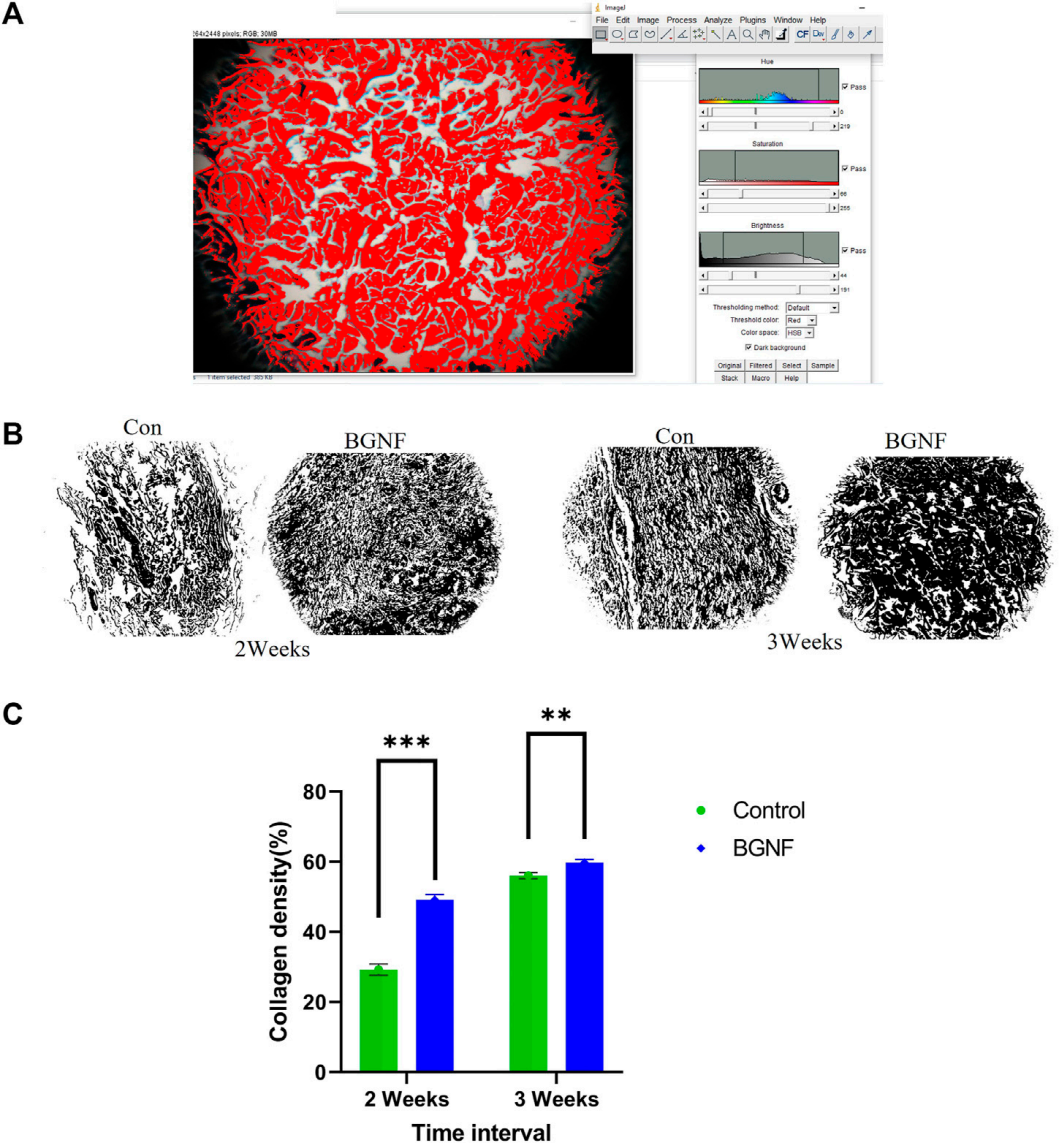
Assessment of Collagen fiber density. **(A)** Collagen density measuring using color threshold method in ImageJ **(B)** Collagen mask of different wound groups at different time intervals (2 and 3 weeks). **(C)** mean percentage of collagen density of control and BGNF-treated groups at 2 and 3 weeks.

Histomorphometric analysis of collagen density of trichrome stained wounds ranged from 49.14% ‐ 59.74% at 2 and 3 weeks time intervals respectively BGnf treated group compared to 29.24% ‐ 56.04% at 2 and 3 weeks time intervals for the control wounds respectively (*p* ≤ 0.00001) indicating higher collagen density in the BGnf treated group of wounds when compared to the control wounds (Figures 8B, C).

**FIGURE 9:**
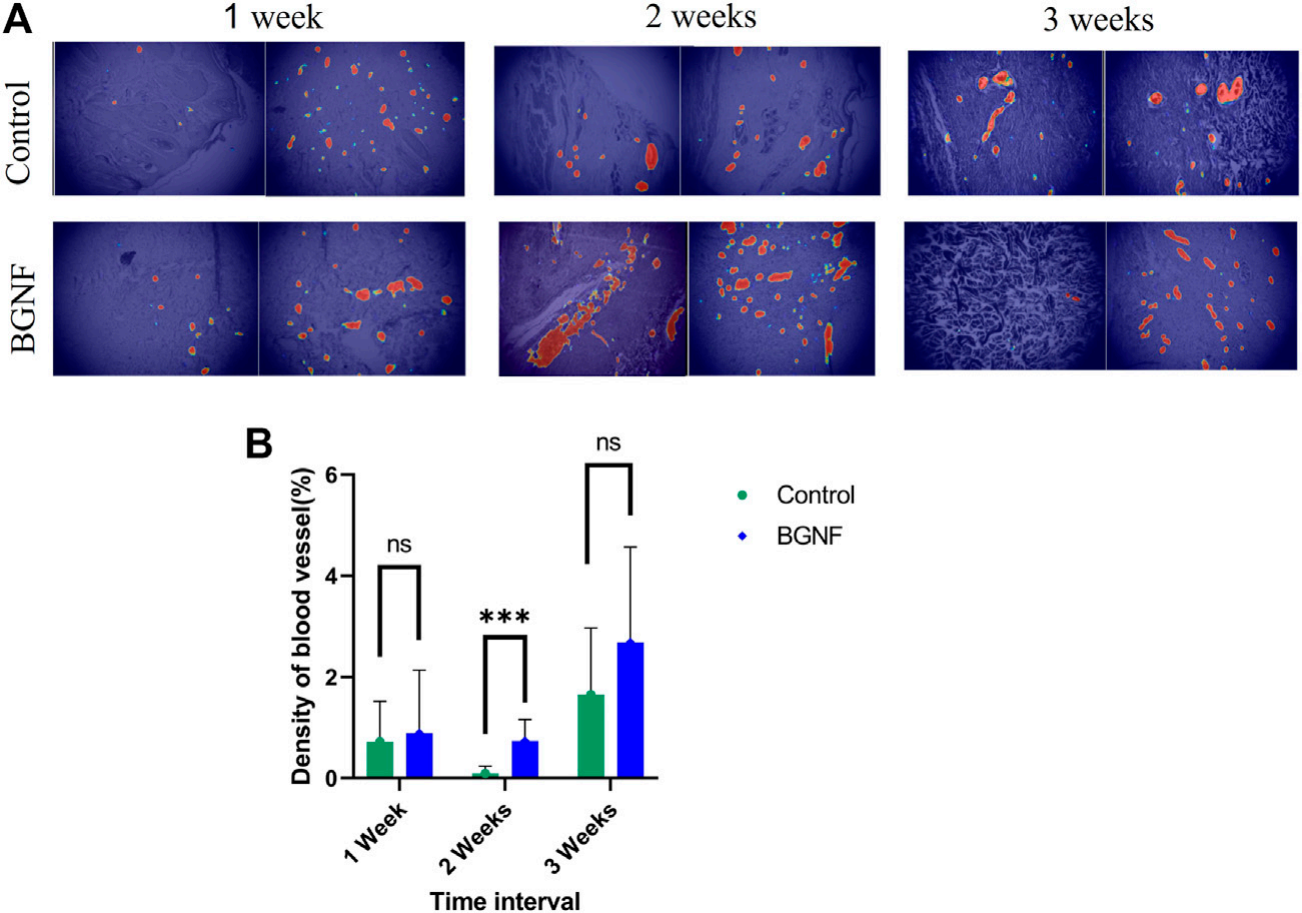
Measuring blood vessels density of untreated and BGNF-treated wounds using IKOSA software. **(A)** Light microscopic images of untreated and BGNF-treated groups processed by the IKOSA software showing blood vessel distribution at different time intervals. **(B)** Mean percentage of blood vessel area of both groups of wounds at different time intervals.

Measuring the mean percentage of total area of blood vessels in the experimental and control wounds at 1, 2, and3 weeks time intervals showed unexpected fluctuation of the blood vessels density values of (0.89%, 0.739% and 2.6%) at 1, 2 and3 weeks time intervals respectively for the experimental groups of wounds. The same was observed for the control group of wounds with blood vessel density values of (0.723%, 0.093% and 1.65%) at 1, 2 and 3 weeks (*p* = 0.608, 0.001 and 0.0517) respectively (Figure 9B). This indicate the higher blood vessel density in the experimental groups at all time intervals when compared to the control group especially at 2 weeks’ time interval where a significant difference was found between groups.

## 4 Discussion

Wound healing is a highly dynamic, organized, and interactive series of events aimed at reestablishing the integrity and function of tissues. Effective management of wounds significantly enhances the speed of healing and the cosmetic appearance of the wounds (Singer and Clark, 1999; Lin et al., 2012). Full-thickness skin wound healing is composed of three overlapping phases of events overall described as inflammation, proliferation, and remodeling (Singer and Clark, 1999). Interruption to any of these three phases may lead to the development of nonhealing chronic wounds which mostly happen in diabetes mellitus (Agren et al., 2000; Lin et al., 2012; Han and Ceilley, 2017). Indeed, the healing of diabetic wounds is sophisticated as a result of vascular, immune function, neurologic and biochemical abnormalities accompanying it (Agren et al., 2000; Han and Ceilley, 2017).

Bioactive glass launched forth a new age in healthcare, laying the foundation for biomaterial-driven regenerative applications (Hench, 2006; Rahaman et al., 2011; Baino et al., 2018). Many different compositions and various types of BGs have been introduced during the last 4 decades to optimize the body’s response to certain therapeutic uses such as bone, neurological, and soft tissue wound healing (AM, 2018; Baino et al., 2018; Cannio et al., 2021). This current work reported the effect of a new formula of borosilicate BG nanofibers composed of ((**1–2) mol% of B**_**2**_ **O**_**3**_ **(68–69) mol% of SiO**_**2**_ **, and (29–30) mol% of CaO**) on full-thickness skin wound healing in a rabbit model. Our results demonstrated that in response to BG, the wound healing rate was significantly shorter than that of the control group. As for the histological evaluation, the BG grafted wounds illustrated significant neovascularization, fibroblast-like cells recruitment, collagen fibers deposition and wound remodeling when compared to the controlled wounds.

Indeed, the regenerative, angiogenic and antibacterial properties of borate-containing bioactive glass put it on top of the biomaterials hierarchy suitable for stimulating wound healing in healthy and immunocompromised conditions (Ma et al., 2014; Naseri et al., 2017; Kargozar et al., 2019). For example, Day and his research group produced borate bioactive glass micro/nanofibers (13–93B3 glass) with a microstructure simulated that of the fibrin clot (Peter, 2011). These fibers could initiate rapid wound healing in diabetic patients whose wounds were resistant to conventional therapies. The group reported that these fibers could initiate angiogenesis and epidermal cell proliferation and migration thus accelerating wound closure. This material has been commercially available since 2017 for veterinary use (Kargozar et al., 2019). Another study conducted by (Lin et al., 2012) reported the same results when using three different types of bioactive glass microparticles which were (SGBG-58S, NBG-58S, and 45S5) loaded on a gel vehicle for full-thickness skin wound healing in diabetic mice model. Moreover, they informed that all used types of bioactive glass have simulated proliferation and activation of fibroblast cells and have accelerated neovascularization. Furthermore, the bioactive glass used was responsible for the production of VEGF and FGF2 which possess an important role in the process of wound healing, especially in diabetes.

Bioactive glass influences all stages of wound healing as it controls and program cells’ proliferation, migration, and apoptosis (Xynos et al., 2000; Hench, 2009; Sergi et al., 2020). As it is generally understood, fibrin clot formation and the inflammatory response mark the initial stage of wound healing (Singer and Clark, 1999; Tsirogianni et al., 2006; Lin et al., 2012). Indeed, Inflammatory cells clear the wound of foreign particles and germs before being extruded with the eschar or phagocytized by macrophages, whose presence indicates that the inflammatory phase is winding down and the proliferative phase is about to begin (Tsirogianni et al., 2006). In the course of our clinical observation, we noticed that starting from 2 days following surgery, a yellow translucent membranoid material developed in the bioactive glass groups and was hardened across time turning to tissue crust. Histological analysis of this crust tissue at 7 days postoperatively showed the presence of many inflammatory cells, BG nanofibers engulfed by phagocytes in addition to different secretions. These observations suggested that bioactive glass nanofibers may have a role in absorbing exudations from the wound at the initial stage of wound healing. In the same context, Lin and his team reported similar clinical and histological observations when using different formulas of BG gel in treating full-thickness skin wounds in diabetic and healthy mice (Lin et al., 2012). The same clinical observation was also reported by Day and his colleagues (Peter, 2011) after a single application of 13–93B3 borate bioactive glass nanofibers to treat non-healed chronic wounds in diabetic patients. They reported that leaving this layer in the wound bed resulted in a beneficial influence on the wound healing cascade. They also reported material disappearance from the wound bed after 1–3 days of application which was another observation reported by our group suggesting the rapid material dissolution and reaction with body fluids. Indeed, this is considered a privilege of borate containing BG over the silicate containing one which was reported to react five times faster with the simulated body fluids (Huang et al., 2006; Jung and de, 2009).

Naturally, the word inflammation may suggest a lousy perspective as inflammation is confused with infection, and inflamed tissues were considered to be bad. However, some inflammation is good and essential for releasing growth factors that trigger cell proliferation moving the wound into the proliferation phase (Tsirogianni et al., 2006; Peter, 2011). Moreover, macrophages recruited during the inflammatory phase play a significant role in all the following stages of wound healing (Peter, 2011; Krzyszczyk et al., 2018). It is well known that the classically activated macrophages have the microbial capacity and secrete high levels of proinflammatory cytokines which consider helpful during the early stages of wound healing. On the other hand, alternatively activated macrophage (M2) is involved in debris scavenging, inflammation resolution, tissue remodelling and wound regeneration (Krzyszczyk et al., 2018). It is considered a transient cell between the inflammatory phase and proliferation phase of wound healing (Dong et al., 2017; Krzyszczyk et al., 2018).

In the same perspective, Dong and his colleague reported that 45S5 BG directed macrophages toward M2 (Dong et al., 2017). They also reported that BG increased the expression of growth factors such as VEGF, IL10 and TGF β while declining the expression of inflammatory cytokines such as IL1β and TNFα (Dong et al., 2017). (Zhao et al., 2015) also reported that Cu dopped and undoped borate BG increased the expression of VEGF *in vitro* and *in vivo* (Lin et al., 2012) also reported that BG-grafted wounds either diabetic or healthy showed a higher rate of fibroblast cells and newly formed blood vessels when compared to the control groups allowing the formation and sustaining of granulation tissues. In the same context, our group reported that Borate BG with formula (**1);** formula **2) mol% of B**_**2**_ **O**_**3**_ **(68–69) mol% of SiO**_**2**_ **and (29–30) mol% of CaO**) increased the expression of VEGF and Collagen I when used to treat the oral mucosal wound in a diabetic rabbit model (Elshazly et al., 2020). We also observed that bioactive glass grafted wounds at 14 days time intervals revealed a higher number of phagocytic cells and fibroblast-like cells that dived from the deep mucosal layers toward the tissue surface. This may suggest the activation of M2 cells and stem cells to take part in the proliferation phase of wound healing even in the presence of diabetic impact on the wound healing cascade. Similar results were clinically and histologically observed during the present study at 7 and14 days time intervals where the BG grafted wounds showed granulation tissue formation that was characterized histologically by a pool of newly formed blood vessels invading the center of the wound following a vertical direction and surrounded by fibroblast-like cells and collagen bundles. We suggest that part of these cells could be related to the ongoing neovascularization process and the formation of the blood vessel walls. This observation was accompanied by the presence of phagocytic cells in the deep subcutaneous layers that surrounded and engulfed the fibers remanent. Granulation tissue formation in BGnf grated wounds in diabetic and healthy patients was also reported by Day and his colleagues, and Simons and his research group as well starting from 7 days time intervals (Peter, 2011; Simons, 2017).

The histomorphometric analysis of the blood vessels in the present study demonstrated higher blood vessel density of the BGNF grafted wounds when compared with the control wounds with statistical significance at 2 weeks time interval. Indeed, angiogenesis is crucial element for all stages of wound healing as blood allows the recruitment of platelets, inflammatory cells, and stem cells to the site of the wound (Singer and Clark, 1999; Ma et al., 2014; Han and Ceilley, 2017; Kargozar et al., 2019). Besides, it provides the newly formed tissue with the required nutrients, growth factors and oxygenation. In addition, it helps in washing tissue sites from the formed waste products and toxins. As previously mentioned, many studies confirmed that BG contributed to the induction of neovascularization at the site of application (Li and Chang, 2013; Lin et al., 2014; Zhao et al., 2015; Baino et al., 2018; Kargozar et al., 2018; Mazzoni et al., 2021). Certainly, this effect is highly related to the chemical composition and the material form. As BG dissolute when contacting the tissue fluids, its leaching ions start to exert their effect and initiate angiogenic differentiation of different stem cells by upregulating genes related to angiogenesis such as VEGF and bFGF (Nzietchueng et al., 2002; Hench, 2009; Peter, 2011; Kargozar et al., 2018). Also, it is well known that silicate ions induce endothelial cell formation and migration leading to acceleration of blood vessels formation (Xynos et al., 2000; Kargozar et al., 2019; Elshazly et al., 2020; Saha et al., 2020). Incorporation of boron ions in BG helps in rapid glass dissolution and therefore allows rapid ion leaching to the surrounding tissue fluid. Moreover, boron ions can increase the translation of mRNAs encoding angiogenesis and wound-healing growth factors including VEGF and transforming growth factor b (TGF-b) (Nzietchueng et al., 2002; Pizzorno, 2015). In addition, the nanofibrous amorphous form of the glass boosts the degradation of the material and gives a suitable environment that mimics the natural ECM which also influences cell behaviour (Peter, 2011; Ma et al., 2014; Deliormanlı, 2015; Xu et al., 2015; Li et al., 2022). In our study, we concentrated on achieving all these targets by preparing bioactive glass nanofibers (360–740 nm) with crosslinked fibers and multiscale porosity as observed by the SEM. Moreover, the incorporation of the boron ions leads to rapid material degradation within 1 week with a higher level of Si & Ca ions that were detected *in-vitro* using SBF allowing controlling of cell genes involved in the wound healing process. Moreover, as previously mentioned the clinical and histological findings and analysis of newly formed blood vessels in BGnf grafted wounds, at 1, 2 and 3 weeks time intervals showed a noticeable rise in neovascularization when compared to the control wounds. Besides that, these findings were confirmed in our previous *in vivo* and *in-vitro* studies (Elshazly et al., 2020; Saha et al., 2020).

The absence of clinical and histological signs of bacterial infection is another observation in the current study. Our previous study has shown the antibacterial properties of the used formula through *in vitro* studying of its antibacterial potentiality (Saha et al., 2020). It has been found that the BG nanofiber extracts exhibited a higher antibacterial efficacy than tobramycin antibiotic against Gram-positive strains *S. Aureus*. Besides, our previous *in vivo* study confirmed the antibacterial properties of the same formula of bioactive glass nanofibers when used for oral mucosa wound healing (Elshazly et al., 2020). Other studies also confirmed the same observation with other BG formulas (Jones et al., 2006; Chandrasekar et al., 2015; Simons, 2017; Drago et al., 2018). Without doubt, ionic dissolution plays a major role in the antibacterial properties of the BG. The contentious leaching of the ions into the surrounding tissues allows for the rise of the alkaline pH leading to the bactericidal effect of the glass (Xynos et al., 2000; Hench, 2006; Jones et al., 2006; Lin et al., 2012; Li et al., 2022).

The wound closure rate of the BG nanofibers treated wounds revealed faster closure when compared with the control group of wounds. Additionally, on the histological level, the BG-treated group showed complete wound reepithelization observed at 14 days’ time intervals. The epithelial layer of the BG wound also manifested with active deeply stained basal *cell layer* indicating ongoing cell division. Indeed, these results are highly correlated with the nanofibrous form of the material that gives the suitable ECM for initiating and accelerating the wound healing cascade (Kim et al., 2006; Li et al., 2014; Deliormanlı, 2015). Moreover, rapid epidermal migration and wound re-epithelialization are the final desired results and it is highly related to all previously mentioned properties of BG as angiogenic potentiality, antibacterial properties, and cell programming. It was reported that bioactive glass can enhance epidermal closure by different mechanisms (Kargozar et al., 2019). One of these mechanisms was mentioned by (Li et al., 2016) who found that 45S5 BG extract activates protein connexin 43 (which allows intercellular gap junction communications) at the gap junctions (Cx43). In skin tissue, this protein is in different sites such as cutaneous vasculature, fibroblasts, dermal appendages as well as the basal and lower spinous cell layers of the epidermis, and its activation prompts wound healing (Coutinho et al., 2003; Wong et al., 2016). Another study conducted by (Yu et al., 2016) reported BG stimulation of fibroblasts to assert essential growth factors such as VEGF, bFGF, and epidermal growth factor (EGF), collagen type I, and fibronectin. The *invivo* observation of the previous study also confirmed the migration of fibroblast cells to the wound bed and the formation of the thick layer of neo epidermis in the BG grafted wounds. In our previous study (Elshazly et al., 2020)., we also observed an increase in the collagen I expression in the BG-treated wounds at 1- and 3-week time intervals when compared with the control groups which coincide with Yu and his team’s findings (Yu et al., 2016). Moreover, the wound healing study conducted by (Zhao et al., 2015) using Cu dopped and undoped borate bioactive glass nanofibers confirmed the valuable effect of angiogenesis and antibacterial properties on speeding up the wound healing rate. The same results were confirmed in the recent study where the BGNF grafted wounds reported higher collagen density at 2 and3 weeks time intervals when compared to the control group.

Despite the disappearance of the material from the wound bed after 1/2 days of application, the wound showed superior clinical and histological manifestations over the control wounds. One reason for that is the existence of material remnants in the subcutaneous skin layer. Another reason may be engulfing of the material by phagocytic cells which might be subjected to the transformation into the M2 cells. Also, we are suggesting that the remaining material during this short period helps in recruiting platelets important to activate the primary steps of wound healing. Also, the presence of the BGnf during the early phase of wound healing was able to stimulate (kick-start) and modulate the inflammatory phase thereby triggering a regenerative cascade of healing rather than a persistent inflammatory one. The nanofiber’s disappearance was also mentioned by Peter (2011) and Simons (2017) as previously mentioned without any notice of the deterioration of the healing rate. This indicates that only a single application of the material will be sufficient to induce and enhance wound healing. This is highly effective from an economic point of view. BGnf is a great grafting material, in our opinion, to be utilized as a therapy for soft tissue deficiencies regularly. It is a low-cost, simple-to-install scaffold. It can also be tailored to the wound region and compressed on the inside. Furthermore, the scaffold is simple to use and can be subjected to multiple applications without any troubles. Similar findings in other research were connected to the simplicity of material application and its cost-effectiveness ((Cannio et al., 2021; Mazzoni et al., 2021)).

The present study is a complementary study based on our previous findings when it comes to the *in vitro* and immunohistochemical characterization of the material effect on wound healing. One limitation of the study was the absence of immunohistochemical characterization to the M1, M2 phagocytic cells to detect their role in the *invivo* tissue regeneration. This limitation will be the target of our next study. Despite that, the present study unveils new findings regarding the suitability of this new bioglass nanofibers formula to be used for the initiation of full-thickness skin wound healing for healthywounds. It also opens new horizons for using this material on the level of clinical trials and improving the quality of life for numerous suffering patients.

## 5 Conclusion

The current work describes the synthesis of a unique BGnf compound and shows how effective it is in hastening the healing of full-thickness skin wounds in a rabbit animal model. The characterization of BGnf *in vitro* shows that it has an ultrastructure that mimics a fibrin clot and an amorphous architecture. Additionally, the BGnf’s ionic dissolution products have a greater rate of degradation, which supports the increased bioactivity of the used formula. BGnf speeds up the healing of full-thickness skin wounds. BGnf accelerating the implantation of collagen fibres and epithelial cell migration to the defect location. Additionally, BGnf provides a sterile wound bed by preventing bacterial invasion of the wounded region. When compared to control wounds, angiogenesis is considerably higher in the BGnf transplanted wounds through the whole healing process duration. BGnf has thus been emphasized in the current investigation as a successful grafting biomaterial for the regeneration of full-thickness skin defects.

## Data Availability

The original contributions presented in the study are included in the article, further inquiries can be directed to the corresponding authors.
